# Azadirachtin-Mediated Responses in the Maize Weevil, *Sitophilus zeamais* (Coleoptera: Curculionidae)

**DOI:** 10.3390/insects16030294

**Published:** 2025-03-12

**Authors:** Herlinda Quintero, Johana Quintero Cortes, Angelica Plata-Rueda, Luis Carlos Martínez

**Affiliations:** 1 Department of Production and Plant Protection, University of Nariño, Pasto 602-7244609, Nariño, Colombia; hquintero17b@udenar.edu.co; 2 Department of Entomology, Federal University of Viçosa, Viçosa 36570-900, Minas Gerais, Brazil; johana.cortes@ufv.br; 3 Department of Biology, National University of Colombia, Bogotá 571-3165000, Distrito Capital, Colombia; rplatar@unal.edu.co

**Keywords:** bioinsecticide, plant secondary metabolites, repellency, stored grain pests, survival, toxicity

## Abstract

Corn is one of the world’s most economically important cereal crops and can be attacked by *Sitophilus zeamais*. The control of *S. zeamais* is mostly based on synthetic insecticides; however, populations of this insect have developed resistance to various chemical compounds. New rational insecticides that prevent the development of resistant populations need to be continuously evaluated. This research aimed to assess the effects mediated by azadirachtin on *S. zeamais* populations in terms of mortality, survival, food preference, and repellency. Azadirachtin is toxic to *S. zeamais* at the larval (LC_50_ = 3.36 ppm), pupal (LC_50_ = 23.0 ppm), and adult (LC_50_ = 37.7 ppm) stages. At all stages of insect development, the survival rate was 99.9% in insects not treated with azadirachtin, decreasing to 29.9%, 44.9%, and 68.5% in larvae, pupae, and adults treated with the LC_50_ of the bioinsecticide, respectively. The preference for untreated corn grains was higher with the LC_25_ and LC_50_ of azadirachtin, while the LC_95_ (209 ppm) repelled adults. Azadirachtin causes lethality in *S. zeamais*, compromising its survival and reducing the feeding preference on grains protected with repellent activity at high concentrations. Therefore, the use of this bioinsecticide may offer an alternative to control *S. zeamais* in the field and post-harvest.

## Simple Summary

The maize weevil, *Sitophilus zeamais*, is one of the most devastating pests of stored grain worldwide. Synthetic insecticides are the primary method of controlling this pest, but they have consequences for human health and the environment. This research investigates the use of azadirachtin-based bioinsecticides as an alternative to noxious chemical molecules. The effects of azadirachtin were evaluated against *S. zeamais* populations, focusing on toxicity, survival, food preference, and repellency. Azadirachtin was toxic to *S. zeamais* at low concentrations in the larval, pupal, and adult stages. The survival of *S. zeamais* decreased with increasing lethal concentrations at all developmental stages. Adult *S. zeamais* preferred grains that were not treated with the bioinsecticide. In addition, higher concentrations of azadirachtin resulted in increased repellency against this insect. The results suggest that azadirachtin has deleterious effects on *S. zeamais* and can be used to control its populations in stored corn.

## 1. Introduction

Cereals are economically important foods and are vital for the food security of the global population. Among cereals, corn (*Zea mays* L.), a plant native to Latin America, stands out [1]. In Colombia, it represents 12% of the cultivated area [2]. Therefore, the preservation of stored grains requires technological processes aimed at ensuring food supply and quality through effective and rigorous post-harvest procedures [3]. In this context, various biotic factors cause the deterioration and destruction of corn grains during storage. Among these factors, insects are particularly significant [4], as they contaminate the stored food with excreta and fungi, making them pests or vectors [5].

Different *Sitophilus* species (e.g., *S. granarius*, *S. zeamais*, and *S. oryzae*) are found in tropical or subtropical regions and are considered as destructive pests of cereals and stored products worldwide [6]. Adults of *Sitophilus zeamais* (Motschulsky, 1855) (Coleoptera: Curculionidae) burrow into healthy grains, where females lay eggs. Subsequently, larvae feed on the endosperm and embryo, leading to the loss of germination and emptying of the seed. Studies show that *S. zeamais* can be a major cause of corn grain loss, reducing their weight by 30–40% at high infestation levels [7]. *Sitophilus zeamais* is polyphagous and can feed on other cereals such as rice, sorghum, and wheat [8].

Synthetic insecticides with neurotoxic activity and from different chemical groups such as neonicotinoids [9], organophosphates [10], and pyrethroids [11] are applied to control stored-product pests. Other insecticides that affect energy metabolism and have a fumigant effect, such as methyl bromide and phosphine [12], have been utilized to reduce high infestations. However, the continuous application of insecticides and increased doses have led to the development of resistant populations to several chemical molecules [13]. Various alternatives to synthetic insecticides are being evaluated due to their side effects on humans and the environment. In this context, natural products derived from plants, consisting of a mixture of secondary metabolites such as mono- and sesquiterpenes, are being used for pest control [14,15,16]. Terpenoids are biodegradable, do not cause environmental pollution, have a low impact on human health, and serve as an alternative to synthetic insecticide use [17].

Neem (*Azadirachta indica* A. Juss., Sapindales: Meliaceae) is important in agriculture because the seeds, leaves, and bark of this tree are used to produce oils and extracts used for pest control [18]. Neem oil is rich in secondary metabolites, particularly azadirachtin, a tetraterpenoid compound used as a biopesticide in agricultural and forestry systems [19]. The mode of action of azadirachtin has not yet been elucidated; however, it acts through contact and ingestion [20]. Studies show that azadirachtin is a potent growth disruptor [21], prevents the synthesis and release of the hormone ecdysone involved in ecdysis, blocks chitin synthesis, and promotes deformities and incomplete molting in immature stages [22]. In addition, azadirachtin causes disruption during mating [23], sterility in adults [24], the impediment of oviposition [25], and an anti-feeding effect [26], and affects digestion [15], as well as repellency in insects [27]. In particular, azadirachtin is known to be an antagonist of 20-hydroxyecdysone (20E) and juvenile hormone (JH), with the ability to modify or suppress hemolymph 20E and JH by inhibiting the secretion of morphogenetic peptide hormones (PTTH) and allatotropins from the corpus cardiacum complex [28]. Also, azadirachtin interferes with the central nervous system (CNS) of insects by inhibiting excitatory cholinergic transmission and partially blocking calcium channels [29].

There are several ineffective chemical molecules used to control *S. zeamais*; however, the impacts of azadirachtin on this insect have not yet been evaluated. Some results suggest that the use of neem oil can have harmful effects on *Sitophilus* species [30,31,32]. We hypothesize that azadirachtin disrupts key physiological processes in *S. zeamais*, leading to lethal and behavioral effects across developmental stages.

This study evaluated azadirachtins’ insecticidal activity against *S. zeamais* developmental stages. This contributes to understanding how this bioinsecticide controls the maize weevil and how it may help to manage synthetic insecticide resistance.

## 2. Materials and Methods

### 2.1. Insects

Under field conditions, *S. zeamais* individuals at different developmental stages were collected from commercial corn crops and used to establish colonies under controlled conditions in the Entomology Laboratory of the University of Nariño (Pasto, Nariño, Colombia). Weevils were placed in BOD (Biochemical Oxygen Demand) climate chambers, set at 25 ± 2 °C, with a relative humidity (RH) of 70 ± 10%, and a photoperiod of 12L:12D (light–dark). Inside the chambers, insect colonies were isolated in glass jars and fed with corn grains. Third-instar larvae and pupae (carefully extracted from the corn grain) and 48 h-old adults of *S. zeamais* were used in the bioassays.

### 2.2. Azadirachtin

The bioinsecticide Neemazal^®^ EC (azadirachtin 1.2 mL L^−1^ active ingredient), manufactured by Fercon S.A. (Yumbo, Valle del Cauca, Colombia), was produced by hydrodistillation and purchased from Parry America INC., (Arlington, TX, USA). The formulation is an emulsifiable, moderately toxic concentrate (toxicological Class III). Neemazal^®^ EC is registered in Colombia by the Colombian Agricultural Institute (ICA), under the Ministry of Agriculture and Rural Development (MADR) (No. 5725), and is internationally certified for utilization in agricultural systems.

### 2.3. Concentration–Mortality Bioassay

Bioassay was conducted in an acclimatized room at 25 ± 2 °C, 70 ± 10% RH, and 12L:12D (light–dark) photoperiod. The bioinsecticide was diluted in 1 mL of distilled water to obtain a stock solution and to prepare six serial dilutions (10, 20, 40, 80, 160, and 320 ppm (*w*/*v*)). The dilutions were used to evaluate toxicity, determine the concentration–mortality relationship, and estimate lethal concentrations (LC_25_, LC_50_, LC_75_, and LC_95_). Distilled water was used as a control. Subsequently, 1 µL of each bioinsecticide dilution was applied topically with a micropipette to the bodies of 90 insects at the third-instar larval, pupal, and adult stages of *S. zeamais*. The insects were individualized in glass tubes (1 × 12.5 cm) and maintained in a climate-controlled chamber, with corn grains provided as food after exposure to the bioinsecticide/control. For each life stage, three replicates of 30 insects were used for six dilutions and a control (*n* = 1890). The experiment was a completely randomized design (CRD) and the number of dead insects at each development stage was recorded after 48 h of exposure.

### 2.4. Survival Analysis

Individuals of *S. zeamais* were exposed to lethal concentrations (LC_25_, LC_50_, LC_75_, and LC_95_) of the bioinsecticide, which were previously estimated in the concentration–mortality bioassay. Distilled water was used as a control. The exposure procedure was similar to that described in the concentration–mortality bioassay. For each life stage, three replicates of 30 insects were used for six dilutions and a control (*n* = 1890) in a CRD. The number of live individuals at each development stage was recorded every 6 h for 48 h.

### 2.5. Adult Food Preference

Bioassay was conducted in an acclimatized room at 25 ± 2 °C, 70 ± 10% RH, and 12L:12D (light–dark) photoperiod. The preference of *S. zeamais* for treated or untreated corn grains with estimated lethal concentrations (LC_25_, LC_50_, LC_75_, LC_95_) of azadirachtin and control (distilled water) was determined in free-choice tests. Adult *S. zeamais* were individualized in a Petri dish (90 mm × 15 mm) with a cellulose filter paper covering the bottom, hereafter referred to as the arena. The arena was covered with microperforated parafilm to prevent insect escape. Two corn grains, one treated with the bioinsecticide and the other untreated, were placed on opposite sides of the arena and an adult weevil was released in the center. Preference was confirmed when the insect continued to feed on the grains for five minutes. For each estimated LC, thirty replicates (30 insects at four LCs, for a total of 120 insects) of untreated/bioinsecticide-treated grains were used in a CRD.

### 2.6. Repellency

**Figure 1:**
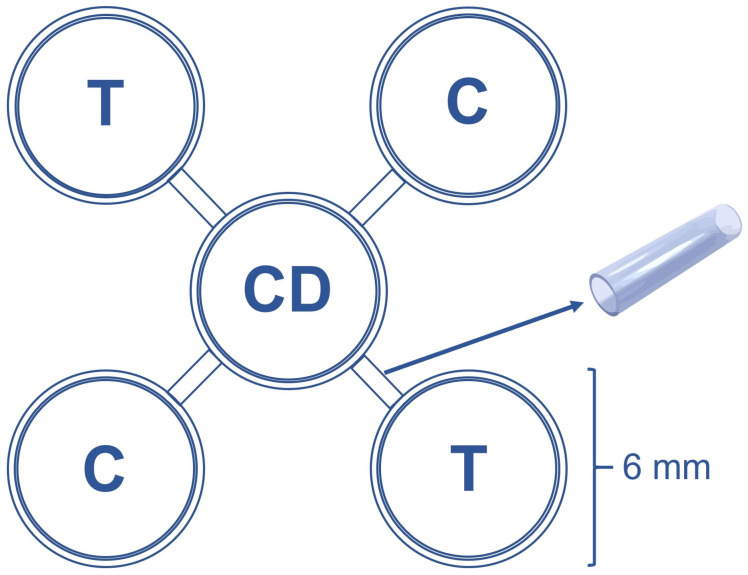
Schematic drawing of four Petri dishes used as an arena, connected to a central Petri dish (CD) with plastic tubes at a 90° angle. Treatment (T) and control (C) were distributed at equidistant points and symmetrically opposed.

Bioassay was conducted in an acclimatized room at 25 ± 2 °C, 70 ± 10% RH, and a 12L:12D (light–dark) photoperiod. Four Petri dishes (60 mm × 15 mm) were used as an arena, connected to a central Petri dish by plastic tubes (2 cm in diameter) at an angle of 90°. The other Petri dishes were distributed equidistantly around the central Petri dish, and two dishes were placed side by side, symmetrically opposite each other (Figure 1). A volume of 1 µL of each estimated lethal concentration (LC_25_, LC_50_, LC_75_, and LC_95_) of azadirachtin was applied in each grain placed on the two opposite plates utilized as treatment, while the two opposite plates with 1 µL of distilled water on each grain represented the control. Twenty corn grains were placed in each Petri dish for the treatment or control. In each replicate of the bioassay, 20 insects were released into the central Petri dish. Four repetitions per treatment/control were used with a total of 80 weevils, and the evaluation time was 30 min, calculating the Repellency Index (RI): IR = 2G/(G + P), where G is the percentage of weevils in the treatment and P is the percentage of insects in the control.

### 2.7. Statistics

Concentration–mortality data were subjected to probit analysis to estimate regression (intercept and slope) and lethal concentration values with 95% confidence limits using SAS v.9.4 software. Time–mortality data were subjected to a Kaplan–Meier survival analysis using GraphPad Prism v.8.1 software. Adult food preference data were subjected to a Student’s *t*-test. Repellency data were evaluated using one-way ANOVA and means were compared utilizing Tukey’s test. An analysis of adult food preference and repellency data was performed using SAS software.

## 3. Results

### 3.1. Concentration–Mortality Bioassay

Concentration–mortality data were suitable for a probit model fit (*p* > 0.05), demonstrating the toxicity of azadirachtin to *S. zeamais* and allowing estimates of toxicological endpoints (Table 1). The results indicated that azadirachtin was most toxic to *S. zeamais* larvae (LC_50_ = 3.36 ppm), followed by pupae (LC_50_ = 23.0 ppm) and adults (LC_50_ = 37.7 ppm). Furthermore, changes in toxicity were observed with the LC_95_ values for larvae (80.3 ppm), pupae (307 ppm), and adults (209 ppm). Mortality in the control was less than 1%.

### 3.2. Survival Analysis

**Figure 2:**
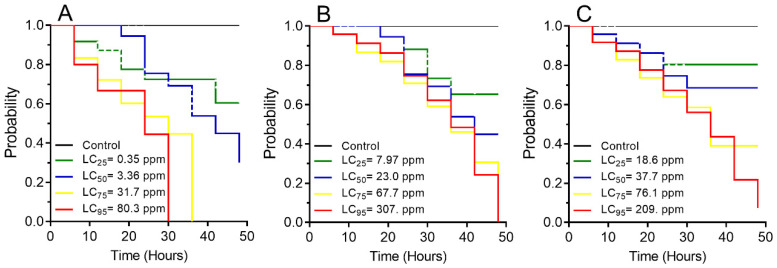
Survival curves of *Sitophilus zeamais* exposed to different lethal concentrations of azadirachtin, estimated by the Kaplan–Meier log-rank test. (**A**) Larva (χ^2^ = 29.68; *p* < 0.001), (**B**) pupa (χ^2^ = 18.11; *p* < 0.001), and (**C**) adult (χ^2^ = 21.05; *p* < 0.001).

The survival of *S. zeamais* was calculated for 48 h after exposure to azadirachtin at different lethal concentrations (LC_25_, LC_50_, LC_75_, LC_95_) and the control (distilled water) (Figure 2). According to the Kaplan–Meier test, the larval survival rates of *S. zeamais* differed significantly with respect to azadirachtin (χ^2^ = 29.68; *p* < 0.001) and decreased from 99.9% (control) to 60.4% with LC_25_, 29.9% with LC_50_, and 0% with LC_75_ and LC_95_. In *S. zeamais* pupae, the survival rate differed significantly (χ^2^ = 18.11; *p* < 0.001) and decreased from 99.9% (control) to 65.3% with LC_25_, 44.9% with LC_50_, 10.2% with LC_75_, and 0% with LC_95_ of azadirachtin. Survival rates in *S. zeamais* adults differed significantly (χ^2^ = 21.05; *p* < 0.001) and decreased from 99.9% (control) to 80.5% with LC_25_, 68.5% with LC_50_, 39.1% with LC_75_, and 7.31% with LC_95_ of azadirachtin.

### 3.3. Adult Food Preference

**Figure 3:**
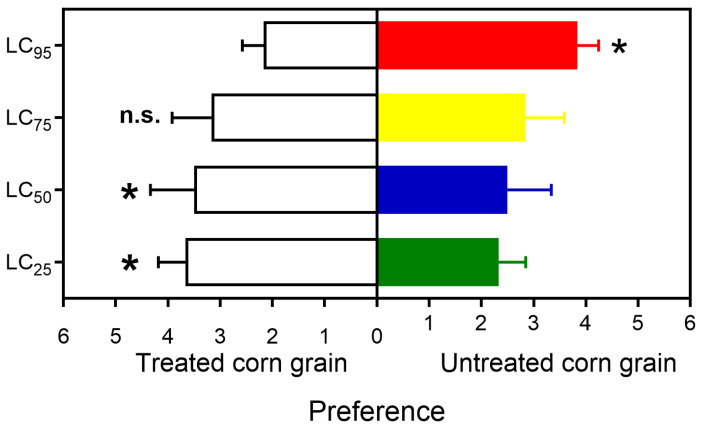
Food preference (Mean ± SEM) in adults of *Sitophilus zeamais* exposed to untreated corn and corn treated with different concentrations of azadirachtin, as assessed by Student’s *t*-test. n.s = not significant, * = significant at 5% significance level.

The adult *S. zeamais* preference for untreated and treated grains varied with different azadirachtin concentrations (Figure 3). The preference for untreated corn grain was higher with LC_95_ (t_1,29_ = 7.071, *p* < 0.001). The preference was similar for untreated and treated corn grains with LC_75_ (t_1,29_ = 0.767, *p* = 0.461), while adults preferred corn grains treated with LC_50_ (t_1,29_ = 2.070, *p* < 0.065) and LC_25_ (t_1,29_ = 4.472, *p* < 0.001).

### 3.4. Repellency

**Figure 4:**
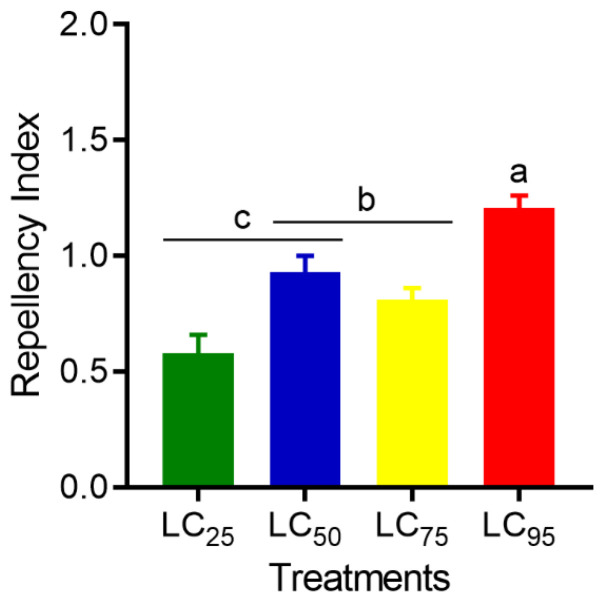
Repellency index (Mean ± SEM) of azadirachtin at different lethal concentrations on adults of *Sitophilus zeamais*. Different letters indicate statistically significant differences between treatments when compared by analysis of variance (one-way ANOVA) followed by Tukey HSD post-test (*p* < 0.05).

Repellency in *S. zeamais* was different after exposure to lethal concentrations of azadirachtin (F_3,3_ = 9.92; *p* < 0.0003). Azadirachtin repelled the highest number of weevils at LC_95_ (RI = 1.21 ± 0.04), followed by LC_50_ (RI = 0.93 ± 0.05), LC_75_ (RI = 0.81 ± 0.07), and LC_25_ (RI = 0.58 ± 0.08) (Figure 4).

## 4. Discussion

In this study, the effects mediated by azadirachtin were evaluated, leading to high mortality, reduced survival, an anti-feeding effect, and repellency across different life stages of *S. zeamais*. Azadirachtin is toxic to *S. zeamais* larvae, pupae, and adults, and exerts a strong effect through topical exposure, resulting in concentration-dependent mortality, as seen in other insects [15,22,25]. Preliminary studies on stored grain pests show that azadirachtin is less toxic to *Tribolium castaneum* (Herbst, 1797) (Coleoptera: Tenebrionidae) with an LC_50_ of 74.2 ppm [33] and *Zabrotes subfasciatus* (Boheman, 1833) (Coleoptera: Chrysomelidae) with an LC_50_ of 2000 ppm [34], compared to the results obtained in this research for *S. zeamais* (LC_50_ = 37.7 ppm). In this context, the developmental stages of *S. zeamais* exposed to azadirachtin concentrations exhibited a loss of mobility, followed by paralysis and death as observed during the experimental bioassay. Although the mechanism of action of this bioinsecticide demonstrates a potent anti-feeding agent, it is possible that azadirachtin also affects the physiological functions of insects through the neuroendocrine and neuronal pathways [28,29]. Exposure to azadirachtin has been shown to interfere with the nervous system of *Nilaparvata lugens* (Stål, 1854) (Hemiptera: Delphacidae) [35], the endocrine system of *Drosophila melanogaster* (Meigen, 1830) (Diptera: Drosophilidae) [36], the reproductive system of *Atta sexdens* (Linnaeus, 1758) (Hymenoptera: Formicidae) [25], and the digestive system of *Anticarsia gemmatalis* (Hübner, 1818) (Lepidoptera: Erebidae) [15]. Although the mode of action is uncertain and the target proteins responsible for the biological activity have not been characterized, azadirachtin caused lethality in *S. zeamais*, suggesting multiple toxic effects on the physiology of this insect. In particular, the results demonstrate that low concentrations of azadirachtin are sufficient to cause toxicity in *S. zeamais* populations and may offer a safe and environmentally friendly alternative for controlling this stored-product pest.

The high variability in *S. zeamais* survival is promoted by the interaction of azadirachtin with penetration through the insect cuticle, leading to the suppression of physiological functions. Short exposures to azadirachtin were sufficient to induce lethality within 30 to 48 h in this insect, demonstrating the rapid action of this bioinsecticide. The rapid action of azadirachtin on insect physiology has also been reported in the respiratory system of *Pieris brassicae* (Linnaeus, 1758) (Lepidoptera: Pieridae) [37], the fat body of *Spodoptera frugiperda* (J.E. Smith, 1797) (Lepidoptera: Noctuidae) [38], and the immune system of *Podisus nigrispinus* (Dallas, 1851) (Hemiptera: Pentatomidae) [39]. On the other hand, the effects compared across developmental stages of *S. zeamais* and the lethal concentrations of azadirachtin occurred at various periods. These time differences are due to the ability of bioinsecticide to interrupt growth and development [40], induce morphological alterations [22], and disrupt the endocrine system [41], leading to the emergence of abnormal individuals. In this research, when comparing the survival of the developmental stages of *S. zeamais*, it was observed that the larva was most susceptible, followed by the pupa and the adult after contact exposure for 48 h, indicating that azadirachtin rapidly reduces the survival of *S. zeamais* and its populations may be more tolerant with age.

The preference of *S. zeamais* adults for corn grains exposed to azadirachtin decreased with higher lethal concentrations. Insect pests can detect the nutritional quality of food [42] and avoid those that are contaminated [43]. For example, *Acrosternum hilare* (Say, 1832) (Hemiptera: Pentatomidae) with a dose of 1520 ppm [44], *Popillia japonica* (Newman, 1841) (Coleoptera: Scarabaeidae) with a dose of 2000 ppm [45], and *Schistocerca americana* (Drury, 1770) (Orthoptera: Acrididae) with a dose of 3000 ppm [46] have been shown to avoid azadirachtin-treated food. In insects, the detection of toxic compounds occurs through chemoreceptors located in the mouthparts and antennae, before contact with contaminated food [47,48]. This enables insects to detect noxious substances that provoke feeding deterrence, inhibit feeding without directly killing the insect [49], and consequently affect digestibility, leading to death through starvation [50]. The present results suggest that azadirachtin has negative effects on the behavior of *S. zeamais*, likely acting as an anti-feedant and deterrent when the insect comes into contact with food treated with this bioinsecticide.

Repellency tests showed that azadirachtin had a greater effect on the behavior of *S. zeamais* with increasing lethal concentrations. Azadirachtin acts as a repellent and is used for the management stored-product beetles such as *Callosobruchus maculatus* (Fabricius, 1775) (Chrysomelidae) [51], *Melanotus communis* (Gyllenhal, 1817) (Elateridae) [27], and *Tribolium confusum* (Jacquelin du Val, 1863) (Tenebrionidae) [52]. In this study, the odor produced by azadirachtin was repulsive to adult *S. zeamais*, and changes in the insects’ behavioral response were observed. These behavioral changes are due to the action of noxious substances that stimulate or reduce insect mobility [53,54,55]. In this context, azadirachtin stimulated the mobility of *S. zeamais*, which rapidly moved towards grains not exposed to the bioinsecticide. During exposure, it is possible that azadirachtin enters the insect through air inhaled via spiracles during the respiratory process and affects the nervous systems, altering behavior, as reported in other insects [56,57,58]. Thus, the behavioral changes at different concentrations of azadirachtin may be due to its effect on sensory modulation through feedback neurons (presynaptic inhibition), which are the action sites of this bioinsecticide. The results suggest that *S. zeamais* was repelled by azadirachtin, exhibiting high behavioral deterrent activity against this insect, indicating its potential to manage stored-product pests.

## 5. Conclusions

Azadirachtin is an effective compound to control *S. zeamais*, causing mortality with the reduced survival of its developmental stages. The low preference for *S. zeamais* observed in this research suggests that adults avoid consuming corn grains protected by this bioinsecticide. Additionally, azadirachtin causes repellency in this insect, with a deterrent effect on adults. For large-scale azadirachtin implementation, its low residual persistence makes chronic conditions unlikely under field conditions. For cost-effectiveness, small doses result in more economical applications that are also more eco-friendly for the environment. Future research in nanotechnology may enhance current formulations of azadirachtin for pest control. Additionally, azadirachtin can be selective in minimizing non-target effects on beneficial insects, such as parasitoids and adult predators. Therefore, azadirachtin-based pesticide could be recommended for the control of *S. zeamais* and incorporated into IPM programs. Overall, azadirachtin is a promising alternative to manage *S. zeamais* populations and can be utilized as a bioinsecticide in the post-harvest handling or storage of corn grains. Although our study demonstrates excellent laboratory results, the validation of azadirachtin’s efficacy in the field or under storage conditions is necessary.

## Figures and Tables

**Table 1:** Lethal concentrations of azadirachtin in different developmental stages of *Sitophilus zeamais* after 48 h of exposure, obtained from probit analysis (df = 5). The chi-square value refers to the goodness-of-fit test at *p* > 0.05.

Developmental Stage	No. of Insects	Lethal Concentration	Estimate Value (ppm)	Confidence Interval to 95% (ppm)	Slope ± SE	χ^2^ (*p*-Value)
Larva	90	LC_25_	0.35	0.22–0.54	1.710 ± 0.13	1.82 (0.10)
	90	LC_50_	3.36	2.26–5.13		
	90	LC_75_	31.7	19.1–58.1		
	90	LC_95_	80.3	36.3–175		
Pupa	90	LC_25_	7.97	5.93–10.1	2.392 ± 0.16	1.34 (0.24)
	90	LC_50_	23.0	18.8–27.9		
	90	LC_75_	66.7	54.3–84.1		
	90	LC_95_	307.	222–462		
Adult	90	LC_25_	18.6	15.5–22.2	3.146 ± 0.20	1.26 (0.27)
	90	LC_50_	37.7	32.3–43.8		
	90	LC_75_	76.1	64.6–91.5		
	90	LC_95_	209.	165–277		

## Data Availability

The original contributions presented in this study are included in the article. Further inquiries can be directed to the corresponding author.
